# Teaching musculoskeletal examination skills to UK medical students: A comparative survey of Rheumatology and Orthopaedic education practice

**DOI:** 10.1186/1472-6920-14-62

**Published:** 2014-03-28

**Authors:** Tim Blake

**Affiliations:** 1 Undergraduate Education Department, South Warwickshire NHS Foundation Trust, Lakin Road, South Warwickshire CV34 5BW, UK; 2 Education and Research Team, Warwick Medical School, University of Warwick, Coventry CV4 7AL, UK

**Keywords:** Musculoskeletal, Undergraduate, Education, Medical, Curriculum

## Abstract

**Background:**

Specialists in Rheumatology and Orthopaedics are frequently involved in undergraduate teaching of musculoskeletal (MSK) examination skills. Students often report that specialty-led teaching is inconsistent, confusing and bears little resemblance to the curricula. The Gait, Arms, Legs and Spine (GALS) is a MSK screening tool that provides a standardised approach to examination despite it being fraught with disapproval and low uptake. Recent studies would appear to support innovative instructional methods of engaging learners such as patient educators and interactive small group teaching.

**Methods:**

This comparative cross-sectional survey evaluates the current state of undergraduate teaching in Rheumatology and Orthopaedics, including preferred teaching methods, attitudes towards GALS, and barriers to effective teaching. An electronic questionnaire was sent to specialist trainees and Consultants in the East and West Midlands region, representing 5 UK medical schools. Descriptive statistical data analysis was performed.

**Results:**

There were 76 respondents representing 5 medical schools. There was a request for newer teaching methodologies to be used: multi-media computer-assisted learning (35.5%), audio-visual aids (31.6%), role-playing (19.7%), and social media (3.9%). It is evident that GALS is under-utilised with 50% of clinicians not using GALS in their teaching.

**Conclusions:**

There is a genuine desire for clinical educators to improve their teaching ability, collaborate more with curriculum planners, and feel valued by institutions. There remains a call for implementing a standardised approach to MSK clinical teaching to supersede GALS.

## Background

Musculoskeletal (MSK) disease has a significant impact on today’s society, both physically and financially [1-5]. What’s more, the morbidity and associated disability of these conditions is projected to increase further, in line with an ageing and heavier population [6].

One would agree that all medical students should receive teaching on MSK clinical skills, and it is hoped that successive generations of doctors would demonstrate confidence and competence when assessing MSK-related conditions. Joint examination should still be regarded as a clinical skill; in fact there can be much reward from identifying pathology with well tried-and-tested techniques. It is important to realise that despite advancements in diagnostic technology, patients still require a thorough and holistic clinical assessment of their condition. Consequently, we have long recognised the need for high quality and enthusiastic teaching of MSK examination skills in the undergraduate arena.

Unfortunately, there are several barriers to delivering this teaching in an effective manner. In spite of the high frequency of MSK disease, the locomotor system is often overlooked and not given the same attention as other bodily systems [7]. Evidently, MSK examination is seen as a neglected skill by practising clinicians [8-10]. This is paralleled by a global inadequacy in clinical skills teaching [11], and lack of confidence on the part of the teacher [12].

Another major obstacle to the enhanced education of MSK examination has been its under-representation in undergraduate curricula [13]. There has been little agreement about what ‘core’ standards are expected of students [8]. This is paralleled by the inter-professional way in which MSK clinical skills are often taught, in that students often report inconsistency and confusion in the way that teaching is delivered. It is discernible that the focus of examination will be different when students are taught by different specialists, although there is consensus that MSK clinical teaching should be simplified and standardised [14].

The advent of the Gait Arms Legs and Spine (GALS) locomotor screen in the 1990s was a major step forward. Not only did it try to simplify locomotor examination for inclusion in the “medical clerking”, it also gave us a novel, practical and standardised way to examine the MSK system, with the aim of detecting important abnormalities and functional disabilities with a high sensitivity and specificity [15,16]. It comprises three screening questions: (1) do you have any pain or stiffness in your muscles, joints or back? (2) Can you dress yourself completely without difficulty? (3) Can you walk up and down the stairs without difficulty? What should follow is a brief screening examination (Table 1). It is inferred that students who are taught the GALS screen as part of the curriculum perform it as confidently as other systems [17]. The same can also be said for doctors [18]. GALS should then be followed by a more detailed assessment of the locomotor system, often referred to as the ‘regional examination’. It is at this stage of the examination that one might expect incongruity between specialists in Rheumatology and Orthopaedics [8].

**Table 1:** GALS screening examination

**Position/Activity**	**Observation**
**Gait**	Symmetry, smoothness of movement (legs, arm swing, pelvic tilting)
	Normal stride length
	Normal heel strike, stance, toe off, swing through
	Ability to turn quickly
**Spine**	
Inspection from behind	Straight spine (no scoliosis)
	Normal, symmetrical paraspinal muscles
	Normal shoulder and gluteal muscle bulk/symmetry
	Level iliac crests
	No popliteal swelling
	No hindfoot swelling/deformity
Inspection from the side	Normal cervical and lumbar lordosis
	Normal (mild) thoracic kyphosis
Inspection from in front	
‘Head on shoulders’	Normal cervical lateral flexion
‘Touch toes’	Normal lumbar spine (and hip) flexion
Palpation from behind	
Press over the midpoint of each supraspinatus	Note any tenderness
**Arms**	
‘Arms behind head’	Normal glenohumeral, sternoclavicular, and acromioclavicular joint movement
‘Arms straight’	Full elbow extension
‘Hands in front’	No wrist/finger swelling or deformity
	Ability to fully extend fingers
‘Turn hands over’	Normal supination/pronation (superior and inferior radioulnar joints)
	Normal palms (no swelling, muscle wasting, erythema)
‘Make a fist’	Normal power grip
‘Fingers on thumb’	Normal fine precision pinch/dexterity
Squeeze across second to fifth metacarpals	Note any tenderness
**Legs**	Normal quadriceps bulk/symmetry
	No knee swelling or deformity (varus/valgus)
	No forefoot/midfoot deformity
	Normal arches
Flex each hip and knee while holding the knee	Confirm full knee flexion with no crepitus
Passively internally rotate each hip in flexion	No pain or restriction
Press on each patella	Note tenderness or effusion
Squeeze across the metatarsals	Note tenderness
Inspect the soles	Note any callosities, reflecting abnormal weight bearing

It is important to note that the number of teaching hours does not always guarantee an improvement in students’ clinical skills; thus in order for MSK specialists to optimise teaching opportunities and offer more efficient learning, they should embrace new teaching strategies and learning styles that are being driven by new medical curricula. More recent evidence-based strategies include use of small group interactive teaching sessions, patient educators and computer-assisted learning (CAL) programmes [11].

## Methods

A questionnaire was developed by TB, then piloted by 7 clinicians at Warwick hospital; made up of medical educators and specialists in Rheumatology and Orthopaedics. A modified version was published as a web survey using the data collection software SurveyMonkey® [19]. This is shown in Table 2. 10 of the questions were compulsory. Questions 6, 7, 9 and 12 allowed respondents to select more than 1 option. Clinicians were asked to select which medical school they provided teaching for. Doctors in the fields of Rheumatology and Orthopaedics at all levels (Specialist Registrar, Consultant, Staff and Associate Specialist, Locum Appointed for Training) were invited to take part in the survey. Invitation was via email; this contained an automated hyperlink and a brief covering letter. Rheumatology specialists in the Midlands region were contacted directly by the author and sent 2 reminder emails during the course of the data collection period. Orthopaedic specialists were contacted by nominated representatives in the West and East Midlands training regions, and also sent 2 reminder emails. Data collection and interpretation was performed using the web-based tool, and further descriptive data analysis was performed using Microsoft Excel. All free text responses were analysed and grouped according to common themes. Responses to the survey were entirely voluntary and without financial or other incentive.

**Table 2:** Questions included in the electronic questionnaire sent to clinicians

**Questions and options included in the electronic questionnaire**	
1.	What is your grade?
	Consultant, SAS, StR, ST3, ST4, ST5, ST6, ST7, ST8, LAT
2.	What is your primary specialty?
	Rheumatology, Orthopaedics
3.	How frequently do you teach musculoskeletal examination skills to medical students?
	Daily, weekly, alternate weeks, monthly, alternate months, less than alternate months
4.	What medical school are you affiliated to?
	Birmingham, Keele, Leicester, Nottingham, Warwick
5.	How would you describe the overall structure of the curriculum at your medical school?
	Traditional, integrated, problem-based, spiral, don’t know
6.	How do you currently teach musculoskeletal examination skills to medical students? (tick all that apply)
	Students practising on peers
	Students practising on instructors
	Students practising on simulated patients
	Students practising on real patients
	Plastic rubber models
	Audio-visual aids
	Role-playing
	Anatomy cadaver lab
	Multi-media computer-assisted learning
	Social media
	Leaflets/Handouts
7.	How would you prefer to teach musculoskeletal examination to improve detection of disease? (tick all that apply)
	Students practising on peers
	Students practising on instructors
	Students practising on simulated patients
	Students practising on real patients
	Plastic rubber models
	Audio-visual aids
	Role-playing
	Anatomy cadaver lab
	Multi-media computer-assisted learning
	Social media
	Leaflets/handouts
8.	Do you teach using GALS (Gait, Arms, Legs, Spine) screen?
	Yes, No
9.	If you do not teach using GALS, why is this? (tick all that apply)
	GALS does not reflect my clinical practice
	It is not incorporated into our local undergraduate curriculum
	I have no experience of using it
	It does not feature in summative assessment of students
	I prefer to have my own individual examination style
	I would rather students spend time on regional examination of the MSK system
	Other (free text)
10.	With reference to GALS:
	I feel confident in performing GALS on patients
	I feel confident in demonstrating GALS to medical students
	I regularly use GALS as part of my patient assessment
	I see GALS being used regularly in the “medical clerking”
	I believe GALS to be an important part of any “medical clerking”
	(strongly disagree, disagree, neutral/unsure, strongly agree)
11.	Is GALS incorporated into the undergraduate curriculum (in your medical school)?
	Yes, No, Don’t know
12.	In your opinion, what are the main barriers to effective undergraduate musculoskeletal examination teaching? (tick all that apply)
	Lack of applicability of teaching techniques to current practice
	Lack of time
	Lack of effective educational tools for teachers
	Lack of a standardised approach to examination
	Lack of interest by students
	Lack of interest by teachers
	Organisational/Institutional
	Other (free text)
13.	What do you see as possible solutions to these barriers, if any?
	Free text
14.	What ways could improve your confidence to teach musculoskeletal examination skills, if any?
	Free text
15.	Any other comments?
	Free text

### Ethics

The survey was reviewed by the local Research and Development team and was deemed to not require Ethics Committee approval.

## Results

There were 76 responses, comprising 49 Consultants (64.5%), 3 StR trainees (3.9%), 4 ST3 trainees (5.3%), 2 ST4 trainees (2.6%), 5 ST5 trainees (6.6%), 6 ST6 trainees (7.9%), 5 ST7 trainees (6.6%), 1 ST8 (1.3%), and 1 LAT (1.3%). No data was received for SAS doctors. 47 (61.8%) of the respondents stated Rheumatology and 29 (38.2%) Orthopaedics as their primary specialty. All midlands’ medical schools were represented by the results: Birmingham 25/76 (32.8%), Keele 8/76 (10.5%), Leicester 18/76 (23.7%), Nottingham 9/76 (11.8%) and Warwick 16/76 (21.1%).

### Frequency of teaching

The majority of respondents, 28/76 (36.8%) were delivering teaching on a weekly basis. 13/76 (17.1%) were teaching on a monthly basis. One clinician was involved in teaching every day.

### Teaching methods

**Figure 1:**
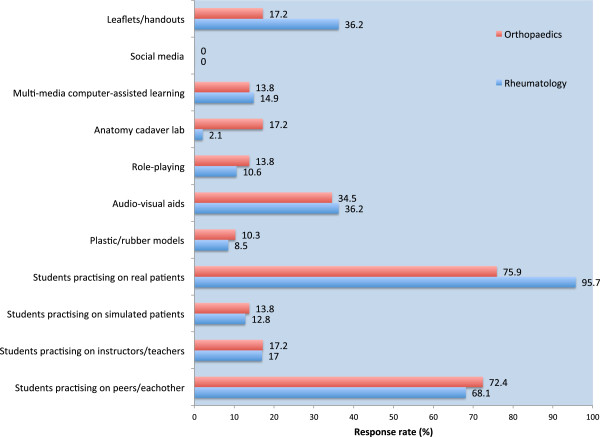
Current ways to teach musculoskeletal examination skills to medical students: Rheumatology versus Orthopaedics.

**Figure 2:**
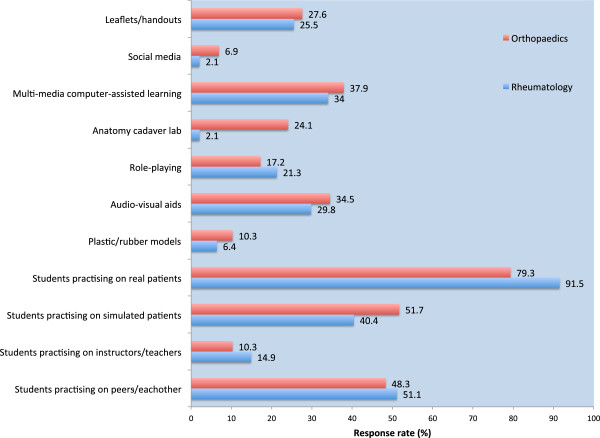
Preferred ways to teach musculoskeletal examination skills to medical students: Rheumatology versus Orthopaedics.

Figure 1 illustrates the comparison in teaching methodologies between the 2 specialties. Overall, there was a partiality for using real patients in clinical skills teaching (67/76, 88.2%), comprising 45 responses from Rheumatology and 22 from Orthopaedics. The next most favoured technique would appear to be the use of peers for practising clinical examination (53/76, 69.7%), consisting of 32 from Rheumatology and 21 from Orthopaedics. Figure 2 goes further to indicate preferred teaching methods for the detection of disease. There was an apparent desire for newer teaching methodologies to be used: role-playing (15/76, 19.7%), multi-media computer-based learning (27/76, 35.5%), audio-visual aids (24/76, 31.6%), and social media (3/76, 3.9%).

### Use of GALS screen

**Figure 3:**
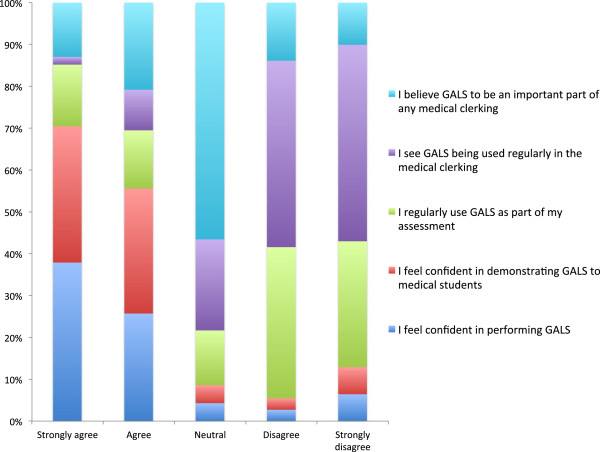
Attitudes towards using GALS in clinical teaching: Rheumatology.

**Figure 4:**
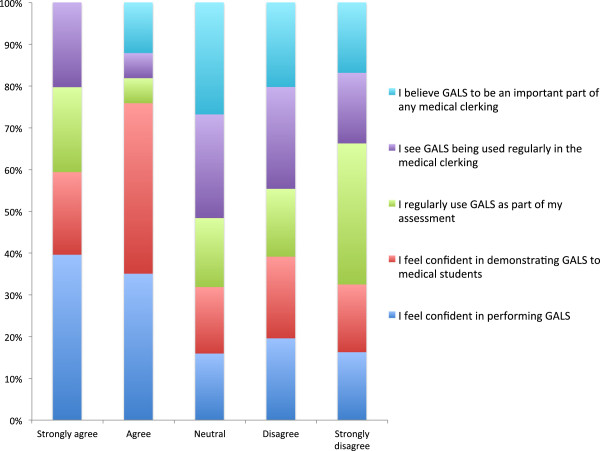
Attitudes towards using GALS in clinical teaching: Orthopaedics.

There was an even split between those clinicians using GALS as part of their teaching (38/76, 50.0%) and those choosing not to teach using this method (38/76, 50.0%). When analysed as separate groups, 36/47 (76.6%) Rheumatologists stated that they use GALS whereas only 2/29 (6.9%) Orthopaedic specialists favoured this approach. With regards to reasons for not incorporating GALS in to teaching, 43/76 (56.5%) clinicians declared that they had no experience of using it, consisting of 14 Rheumatologists and 29 from Orthopaedics. 24/76 (31.6%) stated that they would rather spend time teaching a more thorough regional examination of the MSK system. Aside from this, 24/76 (31.6%) inferred that GALS does not reflect their routine clinical practice, consisting of 10 Rheumatologists and 14 from Orthopaedics. Similarly, 15/76 (20%) of respondents stated that they prefer to have their own individual examination style, therefore GALS is not seen as relevant, made up of 8 Rheumatologists and 7 from Orthopaedics. In Figures 3 and 4 one can appreciate attitudes toward GALS and how these are affected by specialty.

### Undergraduate curriculum

12/76 (15.8%) of respondents were uncertain about the general structure (traditional, integrated, problem-based, spiral) of the undergraduate curriculum at their affiliated medical school. Similarly, 33/76 (43.4%) people were also not sure whether GALS was incorporated into the undergraduate curriculum.

### Barriers to delivering effective teaching

The majority of clinicians (46/73, 63.0%) stated lack of time as the main barrier to giving effective clinical skills teaching. 23/73 (31.5%) felt that organisational and institutional factors were implicated. 22/73 (30.1%) respondents reported that one of the main barriers was the lack of a standardised approach to MSK examination. 19/73 (26.0%) also saw a lack of applicability of teaching techniques to current practice.

### Solutions

There was a definite agreement for hospital teachers to collaborate with medical schools and devote more time to MSK clinical skills throughout undergraduate training. Several respondents wanted to see more contextual teaching reflecting more ‘real world’ scenarios of primary and secondary care. It was suggested that integrated ‘specials’ blocks of the locomotor system could go some way to increasing confidence in this area. Similarly, there was concord for students to be accepted into a firm, giving the implication that students are likely to learn better if they can engage with a consistent learning environment:

“Medical students don’t spend long enough in the department to feel involved in the clinical activity. I hardly see them, even in the city hospitals. I am not sure where the students are”.

“We have medical students who are not even attached to the orthopaedics team, then attend fracture clinic for only one morning”.

There was also praise for designated teaching clinics, where students can be taught in a more realistic and thorough manner, although only if this can be agreed within the funding constraints of the individual hospital. Moreover, there was a loud call to find enthusiastic teachers and to reward those who deliver regular teaching:

“…Acknowledgement of the real value of good teachers rather than the usual lip service paid to teaching. Many appear to think that having students attend clinics is teaching – I would disagree vehemently”.

“Teaching payment directed to only those who actually teach and not absorbed into the general hospital income pot”.

Many Orthopaedic respondents were cynical about undergraduate clinical skills training and felt that too often teaching was merely exam-focused and did little to develop ones interest in Orthopaedics. They did, however, support the use of simulated and computer-assisted techniques to support learning.

### Ways to improve teaching of musculoskeletal clinical skills

Several respondents stated that they were already confident in examination techniques, however there was also a plea for more training of both specialist trainees and consultants in the use of GALS and newer teaching techniques. There was mention of peer observation of teaching and student feedback. With that in mind, it was postulated that this might lead to more consistent and effective teaching:

“I regularly teach hand examination and find that this is certainly something that is consistently taught. I am unsure therefore if I am doing the best for my students!”

“I am reasonably confident but it would be useful to have updates on changes in evidence based examination techniques - I tend only to seek these periodically but otherwise stick to my tried and tested approach”.

It was also seen as imperative for medical schools to provide clinicians and educators with the necessary support for teaching part of the curriculum, and hospital trusts to make provision for designated undergraduate teaching time:

“I would like to see a summary of what is required by the medical school”.

## Discussion

This study sought to compare and contrast current teaching practices between specialists from the fields of Rheumatology and Orthopaedics. At first glance, it would appear encouraging that the responses were not too dissimilar, with the two specialties adopting similar techniques to promote transfer of clinical skills. The majority of respondents favoured the use of real patients and peers to facilitate teaching of MSK clinical skills. Likewise, there was a general call for employing newer more contextual and interactive teaching modalities to drive learning.

A major area of disagreement and deficiency in MSK teaching related to the lack of a standardised approach to the examination. This survey revealed that a large proportion of clinicians preferred to have their own teaching style rather than use the GALS method. This finding echoes the wide variance in examination styles seen in clinical practice across and within specialties, as clinicians adopt individualised ways to detect abnormalities. Unlike other bodily systems, there is no “one size fits all” approach to the locomotor examination; therefore it may come as no surprise that this method has largely been abandoned. Nevertheless, GALS was not intended to be all-inclusive and was developed as part of a two-tier approach to MSK examination, whereby a more a detailed examination could follow on from the initial GALS screen. Although GALS has been validated for use in the undergraduate curriculum [17], its under-representation in day-to-day clinical teaching may reflect a change in attitude towards the screening tool across both specialties, and is likely to explain why the uptake of GALS in the patient clerking has diminished [7,9,18]. Given the fact that no Orthopaedic educators were using GALS to assist in clinical diagnosis, it is unsurprising that only around 7% of respondents utilised this screening tool in their teaching practice. Interestingly, over 75% of Rheumatologists declared that they teach using GALS despite only 21% using this in the day-to-day clinical setting. Walker and Kay inferred that is was at the level of the more thorough ‘regional examination’ that there was disagreement among educators [8], although these findings would stand to contradict this.

These disparities are compounded by the finding that educators are often not familiar with the undergraduate curricula, and consequently there is a desire to increase awareness of learning objectives and how best to teach these. Undoubtedly, clinical educators have a duty to align their teaching methods with the course objectives and intended outcomes, although this notion would appear aspirational when one considers that 16% of clinical teachers are unacquainted with the style of curriculum. If one is to refine the teaching of MSK clinical skills, it is imperative that clinical educators as well as medical school faculty are up to date with the desired competencies for learning.

This study highlighted notable examples of how deficiencies in teaching could be tackled. It was felt that an integrated ‘specials’ block on musculoskeletal disease could help to bridge the gap between different specialties and deliver a more consistent teaching programme. This approach has gained popularity in several UK medical schools, and has been shown to be an effective vector for students’ knowledge, confidence, and satisfaction [20]. Nevertheless, such a heuristic package of teaching requires the motivation and subsequent reward of multiple educators; many of whom are engaged in busy clinical duties. A key theme to emerge was that clinicians were more likely to respond to teaching requests when they had been primed with the course objectives, and given adequate time and financial recompense.

In the current climate, it is crucial that clinical teaching is contextual and reflects the ever-increasing burden of musculoskeletal disease. Competence in MSK examination is essential for all students prior to qualification, although how this is delineated is more complex. Clearly, medical schools should be the drivers for this change in medical education. In the first instance, it has been proposed that a core ‘list’ of MSK regional examination skills should be agreed upon by curriculum planners [8,17,21]; however this is likely to be influenced by the style of medical curriculum and local resources. Medical schools should embrace evidence-based instructional methods of learning and work more closely with clinicians to facilitate meaningful and consistent teaching practice. They should also ensure that the curriculum is reflected in the assessment.

Using a values-based approach to curriculum improvement, it is tempting to rely on traditional methodologies of teaching clinical skills, such as Hays’ apprenticeship model [22]. Fulford et al. remind us that when dealing with value-laden situations, focusing on the values at the expense of evidence can be detrimental to one’s educational development [23]. In the future only time will tell whether using innovative, interactive and contextual ways of teaching will equip medical students with the necessary knowledge, skills, attitudes and behaviour to bring about improvements in clinical diagnosis of MSK disorders. It is likely that advances in medical science and education will further drive development of evidence-based and values-based practice. Thus, one should be open-minded when reviewing the literature on this topic.

Whatever the changes at local organisational or institutional level, one should take individual responsibility for their state of teaching and seek to improve skills accordingly. This may involve attending update courses on aspects of teaching or through regular appraisal. Educators of the MSK system need to be aware that their interpretation of research findings will be coloured by their experiences and values, which may influence decision-making. One solution to these conflicts may be to adopt a collaborative approach in our education practice, akin to the healthcare environment, so that the personal and professional values of several health care workers are taken into account [24]. One such approach to bringing about change in organisations and communities is termed Appreciative Inquiry [25].

### Strengths & Limitations

This is the first time that the musculoskeletal specialties have been compared with respect to undergraduate clinical teaching. It raises important questions about how MSK clinical skills should be delivered in the 21^st^ century, and highlights the need for further studies in this area. This study exhibits several limitations. The fact that only the midlands region was surveyed means that it would be inaccurate to foster any generalisations to the wider community. This survey, in line with other online surveys, is hampered by a low response rate. There was also over-representation of Consultants and Rheumatologists.

## Conclusion

The locomotor system is often seen as complex and difficult to examine. The GALS provided us with a user-friendly, evidence-based and standardised screening tool for the detection of MSK abnormalities. It has been included in global recommendations for a MSK undergraduate curriculum [21]; however GALS is being used less and less in teaching and also clinical practice [18]. The fact that we still do not have uniformity in the way that MSK clinical skills are taught can only add to the confusion and frustration of medical students. In the future, it is hoped that newer evidence-based strategies for teaching will become embedded in curricula and develop our confidence as educators. In an ideal setting, specialists in Rheumatology and Orthopaedics would collaborate more to make clinical skills teaching more contextual and disease-specific. On a positive note this survey demonstrates that these specialties actually have very similar ideas for future clinical skills training, therefore now would seem a good time to define standards and promote curriculum changes.

## Abbreviations

GALS: (Gait, arms, legs and spine) locomotor screen; MSK: Musculoskeletal; CAL: Computer-assisted learning; StR: Specialty Registrar; ST3-8: Specialist trainee; LAT: Locum Appointed for Training; SAS: Staff and Associate Specialist.

## Competing interests

The author declares that they have no competing interests.

## Authors’ information

TB is a Clinical Education Fellow at South Warwickshire NHS Foundation Trust, and is undertaking a Masters in Medical Education (MMedEd) at University of Warwick. He is also a Specialist Registrar trainee in Rheumatology and General Internal Medicine in the West Midlands Deanery.

## Pre-publication history

The pre-publication history for this paper can be accessed here:

http://www.biomedcentral.com/1472-6920/14/62/prepub
